# The Triangular Model of Psychological Stress, Sleep Disorders and Food Addiction in T2DM: An Integrative Review Based on Shared Molecular Mechanisms

**DOI:** 10.3390/nu18111776

**Published:** 2026-05-31

**Authors:** Chunpeng Zhang, Yan Huang, Gaoyang Fu, Xiaoxi Zhang, Daozong Xia

**Affiliations:** School of Pharmaceutical Sciences, Zhejiang Chinese Medical University, Hangzhou 310053, China

**Keywords:** psychological stress, sleep disorders, food addiction, type 2 diabetes, mental health, nutritional psychiatry

## Abstract

The prevalence of type 2 diabetes mellitus (T2DM) continues to rise, and traditional models fail to fully explain its pathogenesis, particularly the frequent co-occurrence of T2DM with mental health disorders. Based on a systematic integration of epidemiological and molecular biological studies, this review organises existing evidence into a “psychological stress–sleep disturbance–food addiction” triangular framework, drawing together observations that have mostly been discussed in isolation. In this model, the three factors form a self-perpetuating vicious cycle through bidirectional interactions, which may synergistically amplify the risk of both T2DM and comorbid mental health conditions via shared molecular pathways. Mechanistically, the model operates through three tiers of pathological amplification: central drive (HPA axis and autonomic imbalance), peripheral effects (glucocorticoid resistance-driven inflammation and metabolic dysregulation), and tissue damage (insulin resistance and β-cell dysfunction). Glucocorticoid resistance serves as the key link connecting central overdrive to amplified peripheral inflammation. The same neuroendocrine and inflammatory pathways are implicated in mood and cognitive disturbances, suggesting a biological basis for the mental–metabolic comorbidity observed clinically. This framework provides an integrated understanding of how psychosocial and dietary factors converge on common biological targets and offers a theoretical foundation for developing integrated nutritional and psychological prevention strategies.

## 1. Introduction

The prevalence of type 2 diabetes mellitus (T2DM) continues to rise, placing a heavy burden on healthcare systems and socio-economic structures, and has become one of the most serious public health challenges of the 21st century [1]. Concurrently, the twofold to threefold elevated prevalence of depression, anxiety and emotional dysregulation among individuals with T2DM suggests that common neuroendocrine and behavioural mechanisms may underlie both mental and metabolic disease [2,3]. Traditionally, the onset of T2DM has been attributed to the interaction between genetic susceptibility and unhealthy lifestyle factors. The convergence of biomedical and social psychological research has broadened our understanding of the aetiology of T2DM. A wealth of evidence suggests that, in addition to traditional risk factors, complex psychological and behavioural factors also play a significant role in the development and progression of T2DM [4].

Among the numerous psychosocial risk factors for T2DM, psychological stress, sleep disorders and food addiction are particularly prominent. Epidemiological studies have found that chronic sources of psychological stress, such as work-related stress, interpersonal conflicts and experiences of discrimination, have been identified as risk factors for T2DM [5,6]. These factors not only contribute to the disease indirectly by promoting unhealthy behaviours but may also directly trigger pathological mechanisms [6]. Concurrently, sleep disorders—including insufficient sleep duration, poor sleep quality and circadian rhythm disruption—are closely associated with impaired glucose tolerance and an increased risk of insulin resistance [7,8]. Existing research confirms that sleep disorders are a strong predictor of new-onset T2DM [9]. Furthermore, the emerging concept of ‘food addiction’—that is, addictive behaviour towards highly processed, high-sugar and high-fat foods—is increasingly recognised as a key explanation for the epidemiological link between obesity and T2DM [10,11]. It should be noted that the term ‘food addiction’ is used here to describe a pattern of compulsive eating behaviour characterised by neuroadaptive changes; while its neurobiological mechanisms bear similarities to those of substance addiction, it is not equivalent to the diagnosis of substance use disorder as defined in the Diagnostic and Statistical Manual of Mental Disorders, 5th Edition (DSM-5). This behavioural pattern involves neurobiological alterations in the brain’s reward system, operating through mechanisms similar to those of substance use disorders, and may ultimately lead to compulsive binge eating [12]. Each of these three factors individually constitutes a high risk for the onset of T2DM, and there is a bidirectional reinforcing relationship between them, which may collectively contribute to the onset and progression of T2DM.

Clinical and epidemiological studies indicate that psychological stress, sleep disorders and food addiction do not occur in isolation; they frequently co-occur, presenting greater challenges for the management of T2DM [13,14]. For example, stress promotes wakefulness, leading to the development of sleep disorders, which in turn trigger the individual’s craving for comfort foods via neuroendocrine responses, thereby fuelling compulsive overeating and altering the reward value of food [15,16]. Conversely, sleep deprivation can exacerbate stress through neuroendocrine responses and disrupt appetite-regulating hormones, leading to increased feelings of hunger and a heightened craving for high-carbohydrate foods [17,18].

Existing research has clearly demonstrated that stress [19,20], sleep disorders [21,22] and food addiction [10,23] are all risk factors for T2DM; however, the vast majority of studies remain limited to single or paired factors. Nevertheless, current research lacks a systematic framework that integrates these three factors, as well as a clear conceptual approach to explain how this behavioural framework synergistically increases the risk of T2DM. Establishing such a framework would help to integrate multidimensional perspectives, deepen our comprehensive understanding of the aetiology of T2DM, and provide guidance for the development of future multi-behavioural intervention strategies. By elucidating these shared pathways, the triangular model also provides a mechanistic framework for nutritional psychiatry—highlighting how dietary behaviour functions as a critical bridge between psychological distress and the combined burden of mental and metabolic disease.

In summary, this review pulls the evidence together into a single framework—the “psychological stress–sleep disturbance–food addiction triangle model”—to give a structure to findings that have until now remained largely separate. These three factors interact and act synergistically to increase the risk of developing T2DM. This paper first outlines the independent mechanisms by which each of these factors contributes to the development of T2DM. It then analyses the bidirectional interactions between them at the molecular and physiological levels. Finally, by integrating the molecular pathways through which these three factors act in concert, we elucidate how this triangular model promotes the onset and progression of T2DM via synergistic effects. This cyclical behavioural triangular theoretical model offers a novel perspective on understanding the role of psychological and behavioural factors in the pathogenesis of T2DM and lays the theoretical foundation for the future development of multi-targeted prevention strategies and personalised interventions.

## 2. Methods

This integrative review used a structured PubMed search. Keywords and MeSH terms covered the core behaviours and possible mechanisms: (‘psychological stress’ OR ‘sleep-wake disorders’ OR ‘food addiction’) AND (“type 2 diabetes”) AND (molecular OR mechanism OR inflammation OR ‘glucagon-like peptide-1’ OR cytokines). No start-date limit was set; the search went through December 2025.

Titles and abstracts were screened for relevance. We included studies that examined at least one of the three behavioural factors and T2DM, prioritising those that addressed molecular, neuroendocrine, inflammatory, or metabolic mechanisms. Both human and animal studies were considered. We excluded work focused solely on type 1 diabetes, gestational diabetes, or other non-T2DM forms, as well as non-English publications.

The selected articles—original research, reviews, and meta-analyses—were synthesised thematically. We identified the independent pathophysiological pathways for each factor, mapped their bidirectional interactions, and integrated the shared molecular networks to build the triangular framework.

## 3. The Independent Pathophysiological Effects of Various Factors on T2DM

Before examining the complex interactions within the behavioural triangle, it is essential to clarify how psychological stress, sleep disorders and food addiction independently increase the risk of T2DM. This will provide a biological basis for the subsequent analysis of their synergistic effects.

### 3.1. Psychological Stress: The Hypothalamic–Pituitary–Adrenal Axis and the Sympathetic Nervous System

Chronic psychological stress affects the body’s metabolism by continuously activating the hypothalamic–pituitary–adrenal axis (HPA axis) and the sympathetic nervous system (SNS). Upon perception of a stressor, the hypothalamus releases corticotropin-releasing hormone (CRH), which prompts the pituitary gland to secrete adrenocorticotropic hormone (ACTH), thereby stimulating the adrenal glands to release glucocorticoids (cortisol) [4,24]. Under chronic stress conditions, persistent overactivation of the HPA axis leads to persistently elevated levels of glucocorticoids [25].

Elevated cortisol has direct metabolic effects. Persistently elevated cortisol levels directly stimulate hepatic gluconeogenesis and induce insulin resistance in skeletal muscle and adipose tissue by interfering with insulin signalling pathways [26,27]. At the same time, activation of the sympathetic nervous system (SNS) leads to the release of noradrenaline. Norepinephrine elevates blood glucose and circulating free fatty acid levels by stimulating glucagon secretion and lipolysis in adipose tissue [28,29,30]. This neuroendocrine environment, characterised by elevated glucocorticoids and catecholamines, is highly conducive to inducing a hyperglycaemic metabolic state, significantly increasing the risk of developing T2DM [31].

### 3.2. Sleep Disorders: From Physiological Dysregulation to Their Impact on T2DM

Sleep disorders are a broad concept encompassing various conditions, including insufficient sleep duration, reduced sleep quality and circadian rhythm disruption. In this review, sleep disorders primarily encompass three types: insufficient sleep duration, poor sleep quality and circadian rhythm disruption. Although these types differ in their neuroendocrine mechanisms, as the existing literature predominantly employs comprehensive sleep indices, this paper does not distinguish between subtypes at this stage.

Research indicates that sleep-related physiological disturbances significantly increase the risk of developing T2DM, and this association is independent of traditional risk factors [7,9]. The underlying mechanisms involve multiple pathways, including neuroendocrine function, appetite regulation, energy metabolism and inflammatory responses.

Sleep deprivation can directly disrupt glucose metabolism homeostasis. Sleep restriction can lead to impaired glucose tolerance and reduced insulin sensitivity, thereby increasing the risk of T2DM [8]. Insufficient sleep reduces the nocturnal peak in growth hormone secretion while elevating cortisol levels, resulting in a state of cortisol excess [32,33]. These alterations in the hormonal environment facilitate hepatic gluconeogenesis and the development of peripheral insulin resistance.

Sleep deprivation indirectly contributes to energy excess by altering the balance of appetite-regulating hormones. Research indicates that sleep deprivation alters the secretion of appetite-regulating hormones: it increases levels of ghrelin, a hunger-stimulating hormone, and decreases levels of leptin, an appetite-suppressing hormone, thereby promoting feelings of hunger and leading to increased energy intake [17]. These hormonal changes directly result in increased hunger and reduced satiety, prompting individuals to consume more calories. A prolonged calorie surplus promotes fat accumulation and weight gain, the latter being an independent risk factor for T2DM in its own right.

Sleep deprivation is also associated with increased oxidative stress and the activation of a low-grade inflammatory state. Clinical studies have observed significantly elevated levels of pro-inflammatory cytokines such as interleukin-6 (IL-6) and tumour necrosis factor-α (TNF-α) in the circulation of sleep-deprived subjects [34,35]. These inflammatory mediators can induce systemic insulin resistance by activating signalling pathways such as nuclear factor-κB (NF-κB), thereby interfering with the normal phosphorylation process of insulin receptor substrate (IRS) [8].

### 3.3. Food Addiction: Dysregulation of the Reward System and Compulsive Overeating

It should be noted that food addiction is not currently a separate diagnosis in the DSM-5. In this paper, the term is used to refer to the pattern of compulsive eating behaviour measured by the Yale Food Addiction Scale (YFAS), which overlaps conceptually with binge eating disorder (BED) but differs in its focus.

The core characteristics of food addiction include compulsive consumption of specific foods, uncontrolled eating and withdrawal symptoms, and are often associated with high-calorie, high-sugar and high-fat foods. Foods with high palatability and energy density can trigger neurobiological responses in the brain’s reward circuitry, and this mechanism bears similarities to substance addiction [36,37]. Long-term consumption of such foods leads to downregulation of striatal dopamine D2 receptors (D2R), thereby reducing the sensitivity of the reward system [12,38,39]. Consequently, the individual must increase their intake to achieve the same level of pleasure, a phenomenon known as reward deficiency. This neuroadaptive mechanism drives individuals to continue binge eating despite adverse consequences, which is a hallmark of addictive behaviour.

From a metabolic perspective, a persistent calorie surplus resulting from excessive intake of sugar and saturated fat promotes ectopic fat deposition, triggers endoplasmic reticulum stress, and induces chronic low-grade inflammation within adipose tissue [40]. These processes may impair insulin signalling and β-cell function, thereby establishing a direct pathway from compulsive eating patterns to a high risk of T2DM [10,41]. Throughout the paper, “food addiction” is used as a working term for the behavioural pattern captured by the YFAS. It is not meant to imply a formal diagnosis equivalent to substance use disorders, and the ongoing debate about its classification should be kept in mind when interpreting the model.

## 4. Bidirectional Interactions: The Vicious Cycle Within the Behavioural Triangle

**Figure 1:**
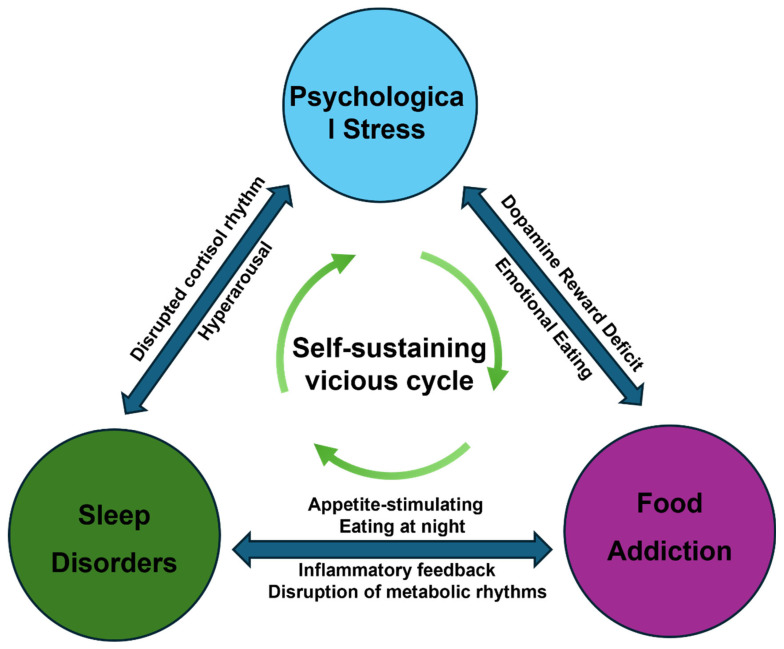
The Triangular Model of Psychological Stress–Sleep Disorders–Food Addiction and Its Bidirectional Interaction Mechanism. Note: The three factors form a cycle through neuroendocrine, metabolic and neural pathways, collectively constituting a self-perpetuating pathological network. The central spiral symbol represents the self-reinforcing nature of this vicious cycle.

The elements of the behavioural triangle do not exist in isolation; there are significant bidirectional interactions between them, whereby they influence and exacerbate one another, ultimately forming a self-perpetuating pathological network. Understanding these complex interrelationships is a key prerequisite for analysing the vicious cycle within the behavioural triangle (Figure 1). Currently, evidence of bidirectional interactions between these three elements is primarily derived from cross-sectional studies and short-term experimental studies, with few long-term longitudinal studies available. The vicious cycle described below is a theoretical inference based on existing evidence and remains to be validated by prospective cohort studies.

### 4.1. The Association Between Psychological Stress and Sleep Disorders

There is a significant bidirectional association between psychological stress and sleep disorders. Psychological stress can cause individuals to remain in a state of prolonged cognitive, emotional and physiological hyperarousal, thereby directly disrupting the physiological processes of sleep [42]. Specific stress-related rumination can lead to delayed sleep onset [43]. Among professionals in high-pressure occupations, sleep problems arising from work-related stress are particularly prevalent [44].

Sleep deprivation in itself constitutes a potent source of physiological and psychological stress. Clinical trials have confirmed that short-term total sleep deprivation significantly amplifies an individual’s negative response to subsequent mild stressors, leading to a marked decline in emotional regulation capacity [45]. Mechanistically, sleep deprivation directly disrupts HPA axis function, leading to abnormal secretion patterns of the stress hormone cortisol and placing the body in a state of heightened physiological stress load [46]. Consequently, a classic vicious cycle develops between psychological stress and sleep disorders: psychological stress disrupts normal sleep structure and quality, while sleep problems in turn exacerbate an individual’s sensitivity to stress. The two are mutually causative and reinforce one another.

### 4.2. The Link Between Psychological Stress and Food Addiction

Psychological stress exerts a significant regulatory influence on eating behaviour. Stress-induced dysregulation of the HPA axis may trigger neurobiological adaptive changes, which in turn promote compulsive overeating [15]. A survey of 1270 adults revealed that high stress levels were positively correlated with the prevalence and consumption of ultra-processed foods [47]. This may stem from stress-induced instinctive behaviour seeking motivational rewards, which in itself constitutes a mechanism for coping with stress [48].

Conversely, consuming ultra-processed foods may also alleviate the negative effects of stress by altering emotional states. The body may enhance positive emotions and reduce negative emotions through the consumption of ultra-processed foods [49,50,51], or individuals may increase their intake in anticipation of the pleasure such foods provide [52]. However, given the weak association between such consumption and emotional states, individuals may need to consume ultra-processed foods repeatedly to maintain their emotional state [53].

Food addiction can also exacerbate psychological stress through various mechanisms. The core neural adaptation underlying food addiction involves downregulation of D2 receptors in the nucleus accumbens and a reduction in the sensitivity of the reward circuitry [54]. This neural mechanism can lead to a loss of pleasure in daily activities, thereby reducing an individual’s coping reserves in response to stressors, causing the same stressors to elicit more intense negative emotional reactions. Furthermore, binge-eating behaviour is often accompanied by feelings of guilt, shame and a sense of loss of control following the episode; these negative self-evaluations are themselves significant sources of psychological stress [55]. Food addiction is highly correlated with obesity, and societal stigmatisation of obesity can independently induce chronic psychological stress, creating a vicious cycle in which obesity leads to increased stress, which in turn further exacerbates binge eating [56]. From a biological perspective, elevated peripheral inflammatory cytokines resulting from food addiction can cross the blood–brain barrier to activate central inflammatory signalling pathways, acting upon the amygdala and prefrontal cortex to exacerbate anxiety and depression-like behaviours [57]. The integrative model proposed by Gupta et al. [58] has highlighted that the stress mediator CRF, the central reward system, and inhibitory mechanisms in the prefrontal cortex collectively contribute to the development of food addiction and obesity, suggesting an overlap in the neuroendocrine mechanisms of the stress and reward systems.

Furthermore, food addiction is considered to be closely linked to obesity and can directly contribute to the development of obesity [59,60]. Obesity itself, along with the associated social stigma, can serve as a persistent source of stress [61,62,63]. Moreover, cross-sectional and prospective studies have shown that excessive consumption of ultra-processed foods is associated with an increased risk of subsequent depression [64]. Furthermore, food addiction may impair adolescents’ mental health, potentially triggering anxiety, depression and suicidal thoughts [65]. Consequently, a vicious cycle emerges between stress and food addiction: stress drives individuals to seek pleasure through eating; food addiction leads to psychological disorders or calorie excess, resulting in obesity; and obesity, in turn, exacerbates stress.

### 4.3. The Link Between Sleep Disorders and Food Addiction

Sleep disorders and food-related addictive behaviours are interlinked through homeostatic regulatory mechanisms and hedonic mechanisms.

Sleep deprivation can increase cravings for high-calorie foods through various mechanisms. As mentioned earlier, insufficient sleep disrupts the balance of appetite-regulating hormones, leading to elevated levels of ghrelin—a hormone that stimulates hunger—while reducing levels of leptin, which suppresses appetite [17]. Neuroimaging studies indicate that sleep deprivation enhances activity in the amygdala during food choice processes while simultaneously weakening the regulatory function of the prefrontal cortex, thereby significantly amplifying cravings for high-calorie foods [66,67,68]. These alterations in brain activity lead to an enhanced anticipation of food rewards and a reduction in self-control [66,68], which in turn promotes a preference for high-calorie foods [66,67]. It is worth noting that the effect of short-term acute sleep deprivation on enhancing the processing of hedonic food stimuli is independent of plasma glucose levels [68]. The neurobehavioural state induced by sleep deprivation makes individuals more susceptible to cravings for high-calorie, high-carbohydrate snacks and fosters the compulsive behavioural patterns characteristic of food addiction [53].

Food-related addictive behaviours can also significantly disrupt sleep homeostasis. Clinical studies have found that individuals with binge eating disorder are three times more likely to experience clinically significant insomnia symptoms than those without the disorder [69]. Binge eating can impair sleep quality, reduce sleep duration and disrupt circadian rhythms [70,71,72]. In particular, binge eating itself is a high-risk factor for insomnia [73]. Mechanistically, changes in eating patterns caused by food addiction may interfere with sleep regulation by affecting hypothalamic function. The central hypothalamic system is easily activated by stimuli from highly rewarding foods [74]. Experiments have shown that stimulation of hypothalamic orexin neurons increases the frequency of transitions from non-rapid eye movement (NREM) sleep and rapid eye movement (REM) sleep to wakefulness [75,76]. Abnormal orexin signalling can lead to sleep instability or circadian rhythm disruption [77,78]. Furthermore, a long-term high-fat, high-sugar diet can induce excessive activation of orexin neurons, increasing the number of nocturnal awakenings and fragmenting sleep architecture [79]. Consuming high-calorie foods at night can also cause sharp fluctuations in blood glucose levels, which, via afferent signals from the vagus nerve, disrupt the circadian synchronisation of the suprachiasmatic nucleus [80]. Obesity associated with food addiction may also induce or exacerbate obstructive sleep apnoea syndrome (OSA), which further impairs sleep quality through intermittent hypoxia and sleep fragmentation [81].

It is evident, therefore, that sleep disorders and food addiction reinforce one another through neuroendocrine, orexin signalling and mechanical factors, creating a bidirectional vicious cycle.

## 5. Together, These Factors Contribute to an Increased Risk of Developing T2DM

**Figure 2:**
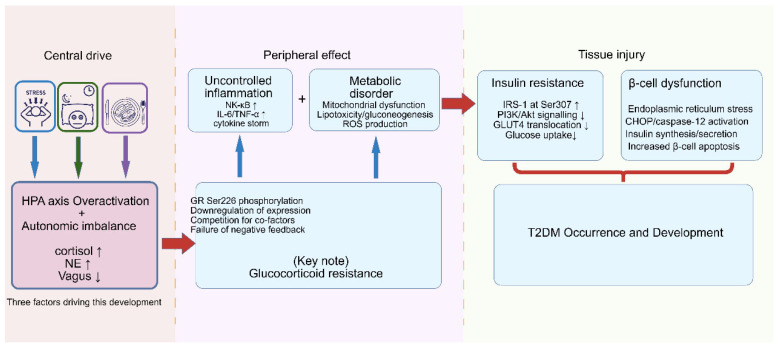
The three-tiered pathological amplification mechanism driving T2DM, as described by the triangular model. Note: Central drive (HPA axis and autonomic nervous system imbalance) transmits pathological signals to peripheral tissues, where glucocorticoid resistance amplifies inflammation and metabolic dysregulation; these peripheral effects ultimately converge on insulin-sensitive tissues and pancreatic β-cells, causing insulin resistance and β-cell dysfunction. This diagram is a simplified view. In living systems, these processes overlap and interact bidirectionally across all levels. ↑ indicates an increase or intensification; ↓ indicates a decrease or suppression.

The three-tiered sequence described below is a conceptual simplification. In the body, central, peripheral, and tissue events run concurrently and are heavily interconnected. When psychological stress, sleep disorders and food addiction coexist, their combined impact on T2DM risk is hypothesised to go beyond the sum of their individual effects, and may arise from cross-amplification among the three factors. The synergistic effects of this triangular framework converge on a common pathological endpoint: the three behaviours mutually reinforce one another, exacerbate metabolic damage, and ultimately jointly drive the core pathological features of T2DM—insulin resistance and β-cell dysfunction. This three-tiered pathological amplification process is illustrated in Figure 2.

### 5.1. Central Drive

The synergistic amplifying effect of the triangular model on the risk of T2DM manifests primarily at the central nervous system level as an imbalance in the interaction between the HPA axis and the autonomic nervous system (ANS). Not only do these three factors activate these two major stress systems independently, but they also maintain a state of chronic central dysregulation through positive feedback loops between them. The mechanisms discussed in this paper primarily concern chronic stress states. Acute HPA axis activation is typically adaptive and differs fundamentally from chronic pathological states.

Chronic psychological stress activates the HPA axis via classical pathways: the paraventricular nucleus (PVN) of the hypothalamus releases CRH, which stimulates the pituitary to release ACTH, thereby stimulating the adrenal cortex to release cortisol [25]. Sleep disorders, particularly sleep deprivation and circadian rhythm disruption, also profoundly affect HPA axis function. Sleep deprivation can elevate nocturnal cortisol levels and reduce sensitivity to negative feedback, leading to abnormal cortisol secretion patterns that mimic and exacerbate a chronic stress state [8,82]. When stress coexists with sleep disturbances, the HPA axis suffers a ‘double blow’: on the one hand, it is continuously driven by ascending arousal signals; on the other, negative feedback inhibitory mechanisms are impaired—such as downregulation of glucocorticoid receptor expression in the hippocampus—resulting in cortisol levels being maintained at elevated levels over the long term.

Whether or not one accepts “food addiction” as a formal diagnosis, the compulsive overeating pattern it captures actively worsens the central drive pathology through at least two routes.

A high-calorie, high-sugar, high-fat diet resulting from food addiction—particularly excessive intake of fructose and saturated fatty acids—can induce peripheral metabolic endotoxemia and inflammation in adipose tissue, leading to elevated circulating levels of the pro-inflammatory cytokines IL-1β and TNF-α [83]. These peripheral signals can directly activate inflammatory signalling pathways in the hypothalamic PVN and amygdala via active transport across the blood–brain barrier, vagal afferents, or the choroid plexus, thereby stimulating excessive activity in CRH neurons [84]. Animal studies have confirmed that hypothalamic microglia in mice on a long-term high-fat diet are in an activated state [85].

The core neural adaptation underlying food addiction is downregulation of striatal D2 receptors and reduced sensitivity of the reward circuitry [38]. This alteration not only drives compulsive eating but also weakens the inhibitory control exerted by the prefrontal cortex over the amygdala and hypothalamus [86]. Impaired prefrontal function causes the HPA axis to lose its crucial regulatory role in responding to stressors, resulting in the same stressor inducing a more intense and prolonged release of cortisol [86]. In other words, food addiction indirectly enhances an individual’s neuroendocrine response to psychological stress by blunting the reward system and weakening impulse control, creating a vicious cycle in which stress leads to food addiction, and food addiction exacerbates the stress response.

Excessive activation of the HPA axis is typically accompanied by a functional reorganisation of the autonomic nervous system, manifesting as persistently elevated sympathetic excitability and relative insufficiency of parasympathetic tone. Both stress and sleep disorders can induce this pattern through increased norepinephrine release and a reduction in the high-frequency component of heart rate variability (HRV) [87,88]. Acute consumption of a high-sugar, high-fat diet can stimulate sympathetic activity [89], while long-term binge-eating patterns are associated with elevated basal sympathetic tone [90].

Autonomic imbalance directly drives peripheral metabolic dysregulation: activation of the hepatic sympathetic nervous system stimulates gluconeogenesis and glycogenolysis, thereby increasing hepatic glucose output [91]. Sympathetic activation of white adipose tissue promotes the release of free fatty acids, exacerbating lipotoxicity and insulin resistance [92]. Impaired function of the vagus nerve-mediated cholinergic anti-inflammatory pathway makes peripheral inflammation more difficult to control [93].

In summary, psychological stress and sleep disorders jointly lead to HPA axis dysfunction and the failure of negative feedback, while food addiction reinforces central drive through two pathways—peripheral inflammatory signalling and the blunting of the reward system—thereby creating a self-perpetuating vicious cycle. The excessive activation of the HPA axis and the imbalance between sympathetic and parasympathetic nervous system function interact with one another, together constituting the core driving centre of the triangular model that transmits pathological signals to peripheral tissues.

### 5.2. Peripheral Effects

Persistent activation of the central nervous system induces and sustains chronic low-grade inflammation and metabolic dysfunction in peripheral tissues via neuroendocrine and autonomic nervous system outputs. These two processes are not independent of one another but reinforce each other through positive feedback loops, together forming a triangular model that drives the peripheral effects of T2DM.

Psychological stress, sleep disorders and food addiction can each trigger peripheral inflammatory responses, and when these three factors coexist, they produce a synergistic amplifying effect. Psychological stress leads to the release of norepinephrine by the sympathetic nervous system, which acts on β-adrenergic receptors (β2-ARs) on immune cells. This activates NF-κB via the cAMP-PKA pathway, thereby upregulating the transcription of pro-inflammatory cytokines such as IL-6 and TNF-α [94,95]. Sleep deprivation and circadian rhythm disruption can similarly activate NF-κB, elevating circulating levels of C-reactive protein (CRP), IL-6 and TNF-α [96,97]. Animal studies have shown that fragmented sleep can induce inflammatory infiltration in adipose tissue and the liver [98]. Excessive intake of saturated fatty acids and refined sugars resulting from food addiction promotes the entry of gut-derived lipopolysaccharide (LPS) into the bloodstream, leading to metabolic endotoxemia [99]. LPS entering the bloodstream activates the myeloid differentiation primary response 88 (MyD88)/NF-κB pathway via the toll-like receptor 4 (TLR4) receptor [100]. Concurrently, a high sugar load promotes the formation of advanced glycation end products (AGEs), which, upon binding to their receptor for advanced glycation end products (RAGE), further amplify inflammatory signalling [101]. When these three factors co-occur, NF-κB is subjected to multiple and sustained stimuli from neural, behavioural and metabolic pathways, resulting in a level of activation far exceeding that induced by any single factor alone.

Under the triangular model, the negative feedback mechanism by which glucocorticoids normally counteract inflammation becomes disrupted. Under physiological conditions, cortisol released following activation of the HPA axis exerts a potent anti-inflammatory effect by binding to the glucocorticoid receptor (GR), thereby inhibiting the transcription of NF-κB and its downstream inflammatory factors [102]. However, under the prolonged influence of the triangle model, this negative feedback loop is disrupted. High-fat and high-sugar loads associated with food addiction activate TLR4 and its downstream c-Jun *N*-terminal kinase (JNK)/inhibitor of nuclear factor-κB kinase β (IKKβ) pathway, inducing phosphorylation of serine residues (such as Ser226) on GR in immune cells. This impedes GR nuclear translocation and DNA-binding capacity, resulting in a significant decline in its transcriptional activity [103,104]. Chronic stress can reduce GR expression levels in the hippocampus and peripheral immune cells via epigenetic modifications of the GR gene promoter [105]. Sleep deprivation for 1–2 weeks can reduce GR mRNA expression levels in the locus coeruleus [106]. NF-κB and GR share transcription co-factors; when NF-κB is overactivated, these co-factors are competitively occupied, indirectly inhibiting GR’s transcriptional function [102]. Ultimately, this leads to a significant weakening of cortisol’s ability to suppress inflammation, despite elevated circulating cortisol levels resulting from the sustained activation of the central drive. This disrupts the dynamic equilibrium between the HPA axis and the immune system. With GR resistance in place, elevated cortisol fails to suppress NF-κB-driven inflammation and may instead fuel it further, stimulating central CRH release and inducing more severe GR resistance. This mechanism may produce peripheral inflammation that is substantially greater than what individual or paired factors would induce, creating an environment that strongly favours the development of insulin resistance.

The triangular model also directly disrupts the metabolic homeostasis of peripheral tissues through multiple pathways, while the accumulation of metabolic by-products in turn amplifies the inflammatory response. With regard to glucose metabolism, elevated central cortisol levels and sympathetic activation enhance hepatic glucose output via hepatic GR and hepatic sympathetic nerves, respectively [107,108]. Sleep disturbances further exacerbate fasting and postprandial hyperglycaemia by elevating cortisol levels and altering growth hormone secretion patterns [109,110]. Compulsive overeating resulting from food addiction increases the amplitude and duration of blood glucose fluctuations [111]. Concurrently, excessive intake of fructose and saturated fatty acids associated with food addiction can induce mitochondrial dysfunction in the liver and skeletal muscle [112]. Fructose metabolism bypasses the regulation of phosphofructokinase, rapidly depletes ATP and generates urate, thereby inducing mitochondrial oxidative stress [113,114]. Long-term high-fat loading leads to a decline in mitochondrial β-oxidation capacity and the accumulation of lipid intermediates (such as diacylglycerol and ceramides) [115,116]. These lipotoxic intermediates can directly activate protein kinase Cθ (PKCθ) and IKKβ, interfering with insulin signalling while amplifying inflammatory responses via endoplasmic reticulum stress [117,118].

Pro-inflammatory cytokines (TNF-α, IL-1β) activate JNK and IKKβ, leading to serine phosphorylation of insulin receptor substrate 1 (IRS-1) and inhibition of its tyrosine phosphorylation, thereby causing insulin resistance [119,120]. Insulin resistance, in turn, promotes fat synthesis and lipid accumulation via compensatory hyperinsulinaemia, further exacerbating mitochondrial overload and the inflammatory state. Concurrently, lipotoxicity and hyperglycaemia themselves act as inflammatory stimuli, maintaining and amplifying inflammation through the activation of NF-κB and the NOD-like receptor family pyrin domain-containing 3 (NLRP3) inflammasome [117].

The triangular model induces glucocorticoid-resistance-driven dysregulation of inflammation and mitochondrial dysfunction-mediated metabolic disorders at the peripheral level via multiple pathways. Insulin resistance and inflammation are causally linked and mutually reinforcing, creating a self-sustaining pathological environment in the periphery. This environment acts directly on insulin-target tissues and pancreatic β-cells, providing the direct preconditions for insulin resistance and β-cell dysfunction.

### 5.3. Tissue Damage

Central drivers and peripheral effects ultimately converge on insulin-target tissues and pancreatic β-cells, leading to insulin resistance and β-cell dysfunction. The core value of the triangular model lies in revealing that when psychological stress, sleep disorders and food addiction coexist, their impact on tissue damage may not be merely additive; rather, it could produce a substantially greater destructive effect than any single factor through cross-amplification of multiple pathways.

#### 5.3.1. Insulin Resistance

Insulin resistance is a central mechanism in the pathogenesis of T2DM, characterised by reduced sensitivity to insulin in the liver, skeletal muscle and adipose tissue. The triangular model drives insulin resistance through the synergistic action of multiple mechanisms.

Persistently elevated cortisol levels originating from the central nervous system induce insulin resistance via two pathways. Cortisol directly inhibits the tyrosine phosphorylation of IRS-1, thereby disrupting post-receptor signalling [121]. Cortisol upregulates hormone-sensitive lipase in adipose tissue, promoting the release of free fatty acids; the latter further exacerbates insulin resistance by activating PKCθ and IKKβ [122]. Norepinephrine released by the sympathetic nervous system activates PKA via β-adrenergic receptors, thereby inhibiting insulin-stimulated glucose uptake [123].

The triangular model leads to sustained elevation of inflammatory cytokines such as TNF-α, IL-1β and IL-6 in peripheral tissues. These cytokines activate the JNK and IKKβ kinases, which directly catalyse the phosphorylation of serine residues on IRS-1 [124]. This abnormal modification impedes the binding of IRS-1 to the insulin receptor and the activation of the downstream phosphatidylinositol 3-kinase (PI3K)/protein kinase B (Akt) pathway, resulting in impaired translocation of the glucose transporter 4 (GLUT4) and reduced glucose uptake [125,126].

Excess free fatty acids and ceramides associated with food addiction accumulate in skeletal muscle and the liver, which can induce a decline in mitochondrial β-oxidation and increased oxidative stress. Partially oxidised lipid intermediates can directly activate PKCθ and IKKβ, further contributing to the inhibition of IRS-1 [127]. Mitochondrial dysfunction also leads to reduced ATP synthesis and increased reactive oxygen species (ROS) production; the latter damages insulin signalling pathways via oxidative stress [128].

It should be noted that food-addictive behaviours lead to excessive calorie intake, and this caloric surplus is itself closely associated with T2DM [129,130]. A high-sugar, high-fat diet can induce insulin resistance and damage pancreatic β-cells [131,132]. Persistently elevated levels of glucocorticoids promote insulin resistance and impair pancreatic β-cell function [27,133].

When the triangular model is established, elevated cortisol levels, activated inflammatory responses and lipotoxicity reinforce one another. Together, these three factors exert multiple pressures on IRS-1 from the endocrine, immune and metabolic systems. The pathological amplification resulting from this triple impact is hypothesised to substantially exceed that of any two factors in combination, and may constitute a core feature of this model.

#### 5.3.2. β-Cell Dysfunction

In a state of insulin resistance, pancreatic β-cells compensate by increasing insulin secretion. However, the triangle model directly damages β-cells through multiple pathways, ultimately leading to the failure of this compensatory mechanism.

Chronic hyperglycaemia and elevated levels of free fatty acids resulting from food addiction jointly induce endoplasmic reticulum (ER) stress in β-cells. β-cells are highly sensitive to endoplasmic reticulum (ER) stress [134]. Sustained activation of ER stress can trigger the pro-apoptotic branch of the unfolded protein response, leading to β-cell apoptosis [135]. Lipotoxicity can also further exacerbate endoplasmic reticulum stress and mitochondrial dysfunction via ceramide synthesis [136].

Peripheral inflammatory cytokines such as IL-1β and TNF-α can act directly on β-cells via their receptors. IL-1β activates the NF-κB and JNK pathways, inducing the expression of inducible nitric oxide synthase, which produces large amounts of nitric oxide, thereby inhibiting the mitochondrial electron transport chain and insulin synthesis [137]. TNF-α, on the other hand, can directly induce β-cell apoptosis [138].

The increased production of reactive oxygen species associated with stress and sleep disorders, combined with the excessive mitochondrial burden caused by food addiction, acts synergistically to disrupt the antioxidant defence system of β-cells. Excessive reactive oxygen species can directly damage mitochondrial DNA, exacerbating energy metabolism disorders and activating apoptotic signalling pathways [139].

Cortisol can directly inhibit insulin secretion via glucocorticoid receptors on β-cells and reduce the expression of key transcription factors such as PDX-1 and MafA, thereby impairing the maintenance of β-cell differentiation and functional maturation [140]. Excessive sympathetic nervous system activation (norepinephrine) inhibits glucose-stimulated insulin secretion via α2-adrenergic receptors, while insufficient parasympathetic tone results in the loss of the vagus nerve’s normal stimulatory effect on insulin secretion [141].

In a state of insulin resistance alone, β-cells can maintain blood glucose homeostasis through compensatory proliferation and increased secretion. However, the triangular model attacks β-cells from multiple dimensions—including glucotoxicity, lipotoxicity, inflammation, oxidative stress and neuroendocrine dysfunction—rapidly exhausting compensatory mechanisms and accelerating the transition from compensatory hyperinsulinaemia to decompensated hyperglycaemia. This process represents a critical turning point in the progression of T2DM from a pre-clinical stage to clinical diabetes.

### 5.4. Physical Inactivity as an Amplifier of the Triangular Model

Although not a vertex of the triangle per se, physical inactivity feeds into and is fed by each element of the cycle. Psychological stress, sleep insufficiency, and compulsive overeating all promote sedentary behaviour through fatigue, anhedonia, and time displacement of physical activity [142,143]. In turn, physical inactivity independently exacerbates HPA axis dysregulation by reducing hippocampal glucocorticoid receptor expression, impairs slow-wave sleep, and dampens striatal dopamine D2 receptor availability, thereby intensifying food cravings and reward deficiency [144,145,146]. This creates a secondary feedback loop in which the triangular model drives sedentary behaviour, and sedentariness further entrenches the neuroendocrine and behavioural disturbances of the triangle.

Conversely, regular aerobic exercise exerts multi-target beneficial effects that directly counteract the shared mechanisms of the model: it enhances glucocorticoid receptor sensitivity and restores HPA axis negative feedback, improves sleep architecture and slow-wave sleep duration, upregulates brain-derived neurotrophic factor (BDNF) to support prefrontal inhibitory control over the reward system, and reduces systemic low-grade inflammation via IL-6-mediated anti-inflammatory cascades and inhibition of NF-κB [144,147]. From a clinical perspective, physical activity therefore represents a uniquely powerful single intervention capable of simultaneously attenuating all three vertices of the triangle.

## 6. Discussion and Future Directions

This review examines the physiological and pathological links between psychological stress, sleep disorders and food addiction, supporting the notion that these three factors form a mutually reinforcing behavioural triangle. This behavioural triangle creates a vicious cycle through a shared network of biological mechanisms, collectively increasing the risk of developing T2DM. These behaviours converge on several key pathways: a dysregulated HPA axis, heightened inflammation, impaired metabolism and an imbalanced autonomic nervous system. The synergistic detrimental effects of these three factors occurring concurrently may substantially exceed the simple sum of the effects of any single factor or any two factors acting in combination.

At the mechanistic level, the triangular model provides a comprehensive pathophysiological explanation for how psychological and behavioural factors act in concert to drive T2DM, through central drive, peripheral effects and the progressive amplification of tissue damage. This is precisely where the model’s core theoretical value lies: when psychological stress, sleep disorders and food addiction coexist, their combined effect on tissue damage may substantially exceed that of any single or dual factor in terms of both intensity and duration.

Patients with T2DM may exhibit one or more behavioural or pathophysiological responses within the behavioural triangle model. Under acute stress, patients with T2DM display an excessive cortisol response, and alterations in the HPA axis may serve as a bridge linking psychological stress to T2DM [148]. The impact of diabetes itself on the central nervous system leads to alterations in neurobehavioural and neurotransmitter function, which in turn trigger sleep disorders [149]. The prevalence of sleep disorders is higher among diabetic patients than in the non-diabetic population [150]. The prevalence of food addiction is significantly higher in T2DM patients than in age-matched control groups [151]. Food addiction is relatively common among newly diagnosed T2DM patients and is associated with high calorie intake [152]. This suggests that there may be a bidirectional reinforcing relationship between the Triangular Model and T2DM: the three factors drive the onset and progression of T2DM, while T2DM itself may in turn exacerbate these three behavioural abnormalities.

Furthermore, once T2DM is established, recurrent hyperglycaemic excursions can generate AGEs, which activate NF-κB signalling via RAGE in the central nervous system [153]. This perpetuates hypothalamic neuroinflammation, further dysregulating the HPA axis and reinforcing the very psychological and behavioural disturbances that constitute the triangular model. This secondary cycle adds another layer of self-reinforcement to the vicious cycle described herein.

The synergistic pathological potential of the behavioural triangle is reflected in its closed-loop structure. Psychological stress can trigger dysfunction of the HPA axis and disruption of the reward system, intensifying cravings for high-sugar, high-fat foods and prompting individuals to continue consuming unhealthy foods in pursuit of dopamine rewards, thereby fuelling food addiction. Sleep disturbances disrupt appetite-regulating hormones, promote night-time eating behaviour, increase opportunities for unhealthy eating, and exacerbate the consequences of food addiction. Conversely, the metabolic disturbances triggered by food addiction can further activate the HPA axis, exacerbating systemic low-grade inflammation, which in turn worsens mood and sleep quality [80,154]. This closed-loop cycle of ‘neuroendocrine imbalance → behavioural dysregulation → metabolic deterioration → renewed neuroendocrine imbalance’ renders the triangular model a self-perpetuating vicious cycle.

**Figure 3:**
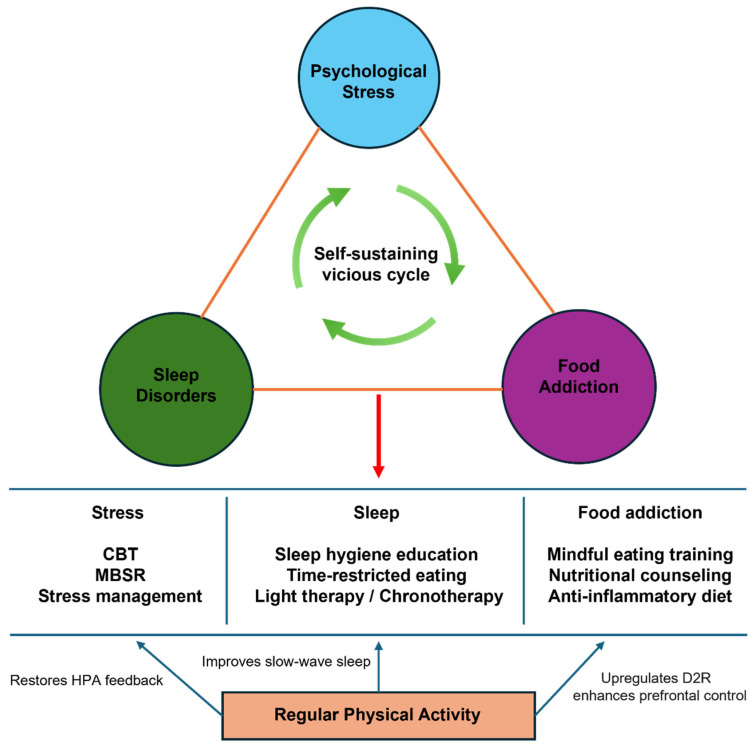
Multi-target intervention strategy based on the triangular model. Note: Targeting the stress–sleep–diet triad through integrated nutritional, psychological and behavioural interventions may simultaneously improve mental and metabolic health outcomes. Abbreviations: CBT, cognitive behavioural therapy; T2DM, type 2 diabetes mellitus.

Given the interconnected nature of the triangle, an intervention targeting a specific behaviour may trigger a beneficial chain reaction throughout the entire system. Clinical studies have provided preliminary evidence that cognitive behavioural therapy can improve depressive symptoms, sleep quality and diabetes self-management behaviours in patients with T2DM while simultaneously reducing short- and medium-term blood glucose levels [155,156]. Mindfulness-based stress reduction therapy can alleviate anxiety, depression and emotional regulation difficulties in obese patients while improving the severity of food addiction [48]. Mindful eating interventions can effectively reduce the glycaemic load in adults with T2DM and improve the intake of trans fats, dietary fibre and sugar [157]. These findings suggest that comprehensive lifestyle intervention programmes should be the preferred option when T2DM patients exhibit behavioural triangle characteristics. Furthermore, targeted drug therapies based on shared molecular mechanisms hold potential for specific patient subtypes. A summary of potential multi-target intervention strategies is provided in Figure 3.

This study has several limitations. Firstly, the proposed behavioural triangle framework is primarily based on clinical observations and mechanistic inferences from longitudinal studies and lacks direct support from large-scale cross-sectional studies. Secondly, while this model primarily explains how the behavioural factors within the triangle drive the initial risk of T2DM, caution is required when interpreting clinical associations due to the possibility of reverse causality: the diagnosis of T2DM itself and the burden of its treatment may also induce or exacerbate psychological stress, sleep disturbances and eating problems. Consequently, this model is more suitable as a theoretical framework for longitudinal prediction and primary prevention, rather than for explaining the progression of symptoms in patients who have already been diagnosed. Thirdly, several confounders deserve attention. Obesity, tightly linked to food addiction, can independently promote inflammation, insulin resistance, and sleep apnoea. Socioeconomic status, physical activity (discussed above), pre-existing depression or anxiety, and medications such as corticosteroids or certain antidepressants may all influence both the behavioural triangle and T2DM risk. Genetic variation in glucocorticoid receptor, dopamine receptor, and circadian clock genes likely modulates individual susceptibility. These factors are not built into the current model and warrant investigation as potential confounders or moderators. Fourthly, there is a need for rigorously designed prospective cohort studies to systematically evaluate the association between the three behavioural factors and T2DM while monitoring the mediating effects of HPA axis function, inflammatory markers, autonomic nervous system activity, and metabolic health indicators. Fifthly, there is an urgent need for further preclinical experiments to elucidate novel biological mechanisms underlying the association between the triangular model and T2DM. It is recommended that experimental designs incorporating controlled stressors, sleep interventions and dietary models be adopted to validate the causal interactions among the three factors and their potential pathways under a unified research protocol. Sixthly, some of the mechanistic evidence cited in this review originates from animal models. Given the differences between rodents and humans in metabolic regulation, neural circuits and behavioural manifestations, caution is required when extrapolating animal study results to human T2DM; future research must validate key findings using human samples.

From a nutritional psychiatry perspective, the triangular model carries three translational implications. First, it identifies stress-driven compulsive overeating—operationalized here under the “food addiction” construct—as a modifiable dietary behaviour that bridges psychological distress and metabolic-mental comorbidity, which makes it a practical target for integrated intervention. Second, the shared molecular pathways—particularly glucocorticoid resistance, gut–brain axis dysregulation, and systemic inflammation—suggest that dietary strategies which limit postprandial glycaemic excursions, such as low-glycaemic index, Mediterranean, or low-carbohydrate dietary patterns, may simultaneously improve metabolic and mental health outcomes by reducing AGEs formation and subsequent NF-κB-driven inflammation. Notably, low-carbohydrate approaches have shown efficacy in ameliorating both insulin resistance and psychological distress in conditions characterised by metabolic–inflammatory overlap, such as lipoedema [158]. These dietary patterns can be combined with omega-3 fatty acid supplementation and microbiome-targeted interventions to synergistically target the gut–brain–adipose inflammatory axis described in this model. Third, it should also be acknowledged that psychological stress in this framework often originates from adverse socioeconomic circumstances, work-related strain, or interpersonal difficulties that lie beyond the individual’s immediate control. While the triangular model elucidates shared biological pathways, effective intervention may require structural and policy-level support alongside individual-level behavioural strategies. Fourthly the framework provides a theoretical rationale for combining nutritional counselling with psychological therapies in the management of T2DM patients with comorbid depression or anxiety. Among lifestyle interventions, regular physical activity warrants particular emphasis, as it concurrently targets the neuroendocrine, sleep, reward, and inflammatory pathways that interconnect the three vertices of the model.

Interventions designed around this triangular framework have not yet been tested. Existing approaches like CBT, mindfulness, or mindful eating mostly target single components. Clinically, one could screen for patients with elevated scores on the Perceived Stress Scale (PSS), Pittsburgh Sleep Quality Index (PSQI), and Yale Food Addiction Scale (YFAS) to identify those fitting the full triangular pattern. Whether a coordinated programme hitting all three vertices outperforms single-focus interventions remains an open question. Future clinical trials should test whether interventions simultaneously targeting the stress–sleep–diet triad can yield synergistic benefits for both glycaemic control and mental health outcomes.

## 7. Conclusions

This review proposes a triangular model linking psychological stress, sleep disorders and food addiction, suggesting that these three factors mutually reinforce one another through a closed-loop causal network, forming a self-perpetuating vicious cycle that collectively drives an increased risk of T2DM. Mechanistically, this model presents a three-tiered pathological amplification process involving central drive, peripheral effects and tissue damage. Glucocorticoid resistance serves as the pivotal link between central over-activation and peripheral inflammatory dysregulation. The combined effect of the triangular model may be substantially larger than the sum of its parts. If validated, this framework could support the development of multi-target strategies based on the triangular phenotype. Future research should validate these causal pathways through prospective cohort studies and mechanistic experiments; breaking this triangular cycle could become a valuable strategy for reducing T2DM burden, though this hypothesis needs testing in prospective cohort studies and randomised trials.

## Data Availability

No new data were created or analyzed in this study. Data Sharing is not applicable to this article.
